# Remimazolam anesthesia for transcatheter mitral valve repair in a patient with mitochondrial myopathy, encephalopathy, lactic acidosis, and stroke-like episodes (MELAS) syndrome: a case report

**DOI:** 10.1186/s40981-022-00528-1

**Published:** 2022-06-01

**Authors:** Atsuhiro Kitaura, Reiko Kosumi, Tatsushige Iwamoto, Shinichi Nakao

**Affiliations:** Faculty of Medicine, Department of Anesthesiology, Kindai University, 377-2 Ono-Higashi, Osaka-Sayama, Japan

**Keywords:** Remimazolam, MELAS syndrome, Mitochondrial diseases, Transcatheter mitral valve repair

## Abstract

**Background:**

Mitochondrial myopathy, encephalopathy, lactic acidosis, and stroke-like episodes (MELAS) syndrome is characterized by cardiac depression, respiratory failure, myopathy, and anesthesia for affected patients is challenging. Although several anesthetics have been safely employed, there are no reports on remimazolam used in those patients.

**Case presentation:**

A 47-year-old male with MELAS syndrome was diagnosed with mitral regurgitation and scheduled for transcatheter mitral valve repair under general anesthesia. Anesthesia was induced with remimazolam and remifentanil (0.3 µg/kg/min). Remimazolam was administered at 12 mg/kg/h until loss of consciousness for approximately 1 min. Anesthesia was maintained with 1.1–1.2 mg/kg/h of remimazolam and 0.1 µg/kg/min of remifentanil without circulatory collapse or severe metabolic acidosis. The tracheal tube was removed in the operating room.

**Conclusion:**

Remimazolam may be a new option for anesthesia for MELAS syndrome patients with depressed heart function.

## Background

Mitochondrial diseases are caused by mutations in mitochondrial DNA (mitDNA) or nuclear DNA (nDNA). Mitochondrial myopathy, encephalopathy, lactic acidosis, and stroke-like episodes (MELAS) syndrome is the most frequent type of mitochondrial disease, characterized by a lack of sufficient aerobic energy metabolism, causing organ dysfunction, especially in organs with a high energy demand such as the nervous system, skeletal and cardiac muscles, kidney, and liver [1, 2]. Avoiding metabolic burden is highly important for patients with MELAS syndrome because it can result in malignant hyperthermia, hypothermia, and resistance to muscle relaxants during surgery [1, 3]. The selection of anesthetic agents is thus important because serious and unexpected complications can occur after anesthetic exposure, although many different anesthetic techniques have been used for MELAS patients [3].

Remimazolam, a new ultra-short-term benzodiazepine, was recently approved [4]. Remimazolam is rapidly hydrolyzed and metabolized primarily by carboxylesterase in the liver. In addition, remimazolam has less influence on hemodynamics than propofol [5]. The properties mentioned above may be advantageous for anesthesia in patients with MELAS syndrome. However, there is no report on remimazolam anesthesia for such patients.

## Case presentation

A 47-year-old man presented with severe heart failure with NYHA III. He had MELAS syndrome due to a point mutation in mitochondrial DNA (3243A → G). His physical features included short stature (155 cm), deafness, renal glomerular lesions, neurological disorders (lower limb muscle weakness, weakness of tendon reflex, and bilateral pathological reflex positive). The manual muscle testing of his lower limbs was three-fifths. He spent his daily life in a wheelchair. Atrophy of both the cerebrum and cerebellum, and small old cerebral infarction were observed on magnetic resonance imaging. His mother and sisters had the same symptoms but were not definitively diagnosed.

Preoperative laboratory test revealed a normal serum creatine kinase level (207 U/L), anemia (hemoglobin 12.0 g/dL), increased serum creatinine (8.6 mg/dL), and lactic acid (3.68 mmol/L) levels. He underwent hemodialysis 3 times a week because of renal failure. Electrocardiography revealed Wolff-Parkinson-White syndrome, and cardiac ultrasonography demonstrated moderate mitral valve regurgitation (MR), low left ventricular ejection fraction (LVEF 0.3), and left ventricular hypertrophy (left ventricular posterior wall dimensions 13 mm). His coronary arteries were intact. Oxygen therapy, diuretics, and the administration of 3 µg/kg/min of dobutamine were started after admission. However, as these treatments were ineffective, transcatheter mitral valve repair using MitraClip (Abbott Vascular, Chicago, IL, USA) under general anesthesia was scheduled.

**Fig. 1:**
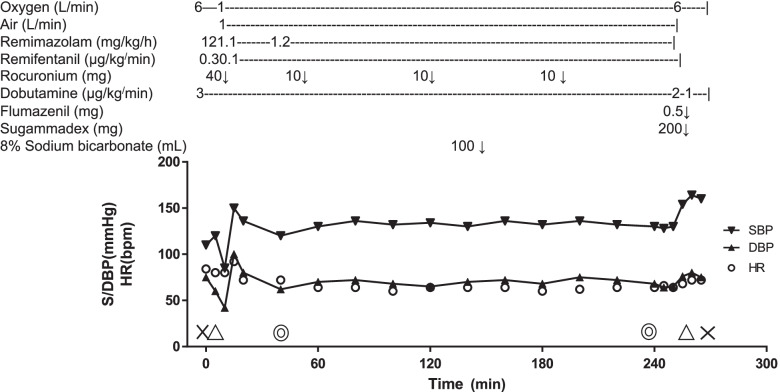
The anesthesia chart. Blood pressure and heart rate were maintained with dobutamine 3 µg/kg/min, which was started preoperatively. SBP: systolic blood pressure, DBP: diastolic blood pressure, HR: heart rate. X: start and end of anesthesia, ∆: tracheal intubation and extubation, double circle: start and end of surgery

On the day of surgery, no premedication was administered, and dobutamine infusion was continued at 3 µg/kg/min until extubation after surgery. In the operating room just before the induction of anesthesia, his blood pressure was 100/68 mmHg and heart rate was 88 bpm. In addition to routine monitoring, a radial artery catheter was placed to monitor BP. His body was warmed using a warm air heating device (Bear Hugger^®^, 3 M Company, Saint Paul, MN, USA) and maintained at 36.0–36.4 °C to prevent hypothermia. Anesthesia was induced with remimazolam and remifentanil (0.3 µg/kg/min). Remimazolam was administered at 12 mg/kg/h until loss of consciousness for approximately 1 min. The trachea was intubated with the aid of rocuronium (0.6 mg/kg). Anesthesia was maintained with 1.1–1.2 mg/kg/h of remimazolam and 0.1 µg/kg/min of remifentanil. The doses of remimazolam were adjusted to bispectral indices ranging from 40 to 60. Dobutamine at 3 µg/kg/min was continuously infused intravenously until he was transferred to the postoperative intensive care unit (ICU). Transesophageal echocardiography (TEE), Flo Trac ™ sensor (Edwards Lifesciences, Irvine, CA, USA), and Edwards oximetry CV catheter were used during transcatheter mitral valve repair. TEE findings after induction of general anesthesia were following: LVEF was 0.3, mitral valve annulus long diameter was 50 mm, short diameter was 45 mm. MR was central jet from A2/P2, and vena contracta width of MR: 6 mm. The results of arterial blood gas examinations are shown in Table 1. Bicarbonate Ringer’s solution with 1% glucose added was administered intravenously. During surgery, 100 mL of 7% sodium bicarbonate was administered when metabolic acidosis progressed. The anesthetic chart is shown in Fig. 1.

**Table 1:** Blood gas data. During anesthesia, metabolic acidosis gradually progressed and was improved by the administration of sodium bicarbonate. Lactic acid and blood glucose levels did not significantly fluctuate

	20 min (post-induction)	50 min (The operation was started)	120 min	150 min	180 min	210 min	240 min
pH	7.465	7.372	7.363	7.504	7.476	7.477	7.506
PaCO_2_ (Torr)	22.7	29.8	26.1	30.5	33.3	30.7	28.7
Base Excess (mEq/L)	-5.6	-6.9	-9.3	1.5	1.5	-0.2	0.4
Blood Glucose (mg/dL)	117	100	90	128	122	108	119
Lactate (mmoL/L)	1.7	1.5	1.2	1.6	1.9	1.6	2
Hemoglobin (g/dL)	11.7	11.31	10	10.4	10.6	9.6	10.4

MitraClip (1 clip via the right femoral approach) implantation was performed without complications. When manual pressure hemostasis at a catheter insertion site was started, remimazolam infusion was stopped. Approximately 10 min after the discontinuation of remimazolam, all surgical procedures were completed. Then, 0.5 mg of flumazenil and 4 mg/kg of sugammadex were administered. One minute after flumazenil administration, the patient regained consciousness and spontaneous breathing, and was extubated. The patient exhibited good arousal and had no pain or shivering. Dobutamine was discontinued in the operating room due to stable hemodynamics. The anesthesia time was 264 min and the operation time was 187 min. The amount of infusion during anesthesia was 920 mL. There was no transfusion. During anesthesia, there was no arrhythmia event. After anesthesia, there were no adverse events such as re-sedation and desaturation. The day after the operation, he left the ICU and started rehabilitation. Five days after the operation, he was discharged.

## Discussion

Mitochondrial dysfunction leads to insufficient aerobic energy metabolism, causing organ dysfunction, especially in organs with a high energy demand such as the nervous system, skeletal and cardiac muscles, kidney, and liver [2]. In MELAS syndrome patients, the serum lactate level is often high due to metabolic acidosis. The goals of anesthesia management are maintaining an adequate oxygen supply to systemic organs. As the patient had poor cardiac function, intraoperative cardiac index (CI) and ScvO_2_ were monitored. CI > 2.2 L/min/m^2^ and ScvO2 > 65% were maintained during anesthesia. As a result of the careful anesthesia management, the increase in lactic acid during anesthesia was suppressed (Table 1).

As some general anesthetics affect mitochondrial function, anesthesiologists should avoid unsuitable anesthetics. In particular, propofol is known to suppress the respiratory chain [6]. Propofol, which is used as a lipid emulsion for clinical use, may be detrimental for patients with fatty acid oxidation disorders [7]. According to two case series on anesthesia for patients with mitochondrial disorders, including MELAS syndrome, propofol can be safely used because inhibition of the respiratory chain in the mitochondria is weak [1], but the number of reported cases was less than 20 [8, 9]. It is therefore safer to avoid the continuous administration of propofol in patients with mitochondrial disorders because mitochondrial dysfunction is involved in propofol infusion syndrome [10]. Sevoflurane is the most common agent for the anesthesia management of children with mitochondrial disorders [11]. However, it should be noted that inhaled anesthetic agents also inhibit the respiratory chain complex. Sevoflurane inhibits the respiratory chain, causing ATP synthase reversal in vitro [12]. Midazolam, opioids, dexmedetomidine, and local anesthetics were demonstrated to be safe for MELAS syndrome patients [8, 13].

Remimazolam is a new ultra-short-acting benzodiazepine anesthetic. To our knowledge, there have been no reports of remimazolam use in a patient with MELAS syndrome. However, as midazolam, a benzodiazepine derivative, was safely used in MELAS syndrome patients [11], we hypothesized that remimazolam can also be safely used for these patients. Remimazolam may be suitable for patients with depressed cardiac function, such as in MELAS syndrome, because it causes less circulatory depression than propofol [5]. In addition, its lesser impact for circulation seems to be beneficial for MitraClip procedure. That is because the MitraClip procedure required a limited preload and high positive end-expiratory pressure (up to about 15 mmHg) is beneficial for a surgeon to perform the MitraClip procedure more easily and safely. Indeed, remimazolam maintained stable hemodynamics in our patient without uncontrollable lactic acidosis. Furthermore, remimazolam has an antagonist, flumazenil, with the same duration of action, enabling rapid and reliable arousal, which is significantly important for anesthesia in patients with neurological disorders. The patient awakened approximately 1 min after flumazenil administration.

In conclusion, a patient with MELAS syndrome who underwent MitraClip® implantation was effectively managed with remimazolam anesthesia. Remimazolam may be a new option for anesthesia in MELAS syndrome patients with depressed heart function.

## Data Availability

Not applicable.
